# A Join Point Survival Model for Brain Tumor Patients

**Published:** 2011-12

**Authors:** Dimitris Vovoras, Frank D. Vrionis, Chris P. Tsokos, Keshav Prokhel

**Affiliations:** 1 *Department of Mathematics and Statistics, University of South Florida, USA;*; 2 *Spinal and Skull Base Oncology, H. Lee Moffitt Cancer Center, USA*

**Keywords:** brain tumor, Cox-PH, joinpoint, SEER, spline, survival

## Abstract

To investigate the relationship between medical improvements and the survival experienced by the patient population, it would be useful to find out when and how much the cancer treatment breakthroughs and early diagnosis have significantly improved the prognosis of brain cancer patients. A join point model facilitates the identification of trends with significant change-points in survival; the main goal of such a model would be to find out when cancer survival starts exhibiting a pattern of improvement. The model will be applied to grouped relative survival data for major cancer sites from the ‘Surveillance, epidemiology and end results’ program of the National Cancer Institute.

## INTRODUCTION

In studying trend data, joinpoint and more general spline models have been used to model the progress of cancer rates, and indicate the time points at which the measures experience a significant change. Joinpoint models have been used for incidence data, as well as mortality rates (12, 13). However, analyzing cancer incidence and mortality is not always enough to understand the benefits of medical breakthroughs in cancer, as it does not provide information on the situation of the patients during their lifetime after diagnosis. In this study we will focus our attention in modeling trends in brain cancer survival by introducing joinpoints following the calendar time of diagnosis using the Surveillance, Epidemiology and End Results (SEER) data sets. The data sets include collected information on incidence, prevalence and survival from specific geographic areas representing 28 percent of the US population (1, 9).

It is essential to model the trend of survival at the population level; tracing the change in survival patterns over time, we can evaluate the effort in improving the survival chance of cancer patients. Here, we consider incorporating a joinpoint model into a population level for capturing possible changes in survival trend. Besides treatment, survival may also be affected by the introduction and dissemination of new screening techniques and other prevention activities.

The survival trend may not have such a big increasing or decreasing pattern as we observe for incidence or mortality, but as discussed in (8), rates usually improve dramatically after the introduction of an effective treatment, and then level off after the dissemination of the cancer treatment has been fully realized to the population, indicating a possibility of the presence of multiple change points in the survival function.

This paper is organized as follows. In section 2 the relevant models and procedures are introduced. In Section 3, we perform the study by presenting the models and assess the performance of medical breakthroughs in brain cancer survival data. Section 4 includes discussion and future research problems.

## METHODS

If *x* is the calendar time and *t* is the survival time from diagnosis to death, we assume that the hazard rate of dying at time *t* follows a proportional hazards model with

(a) $$\lambda \left(t|x\right)=\lambda_{0}\left(t\right)\exp\left\{h\left(x\right)\right\}$$

where *λ_0_(t)* is the baseline hazard and

(b) $$h\left(x\right)=\beta x+\sum_{k-1}^{K}\delta_{k}{\left(x-\tau_{k}\right)}^{+}+\gamma^{'}z$$

will indicate the trend in survival with respect to calendar year of diagnosis *x*. Here *u*^+^ = *u* if *u*>0 and 0 otherwise and *z* will be a vector of other covariates, e.g. race, sex. The τ_1_,..,τ_k_ are called joinpoints because the hazard function *h(x)* has different slopes before and after the joinpoints while continuous. Under the formulation of (b) there are a total of (K + 1) segments, and for the k-th segment the slope coefficient is $\beta_{k}=\beta +\sum_{l=1}^{k-1}\delta_{l},k=1,...,K+1.$ In summary, the baseline hazard is dependent only on *t*, the survival time after diagnosis, while the hazard function is conditional on *z, t*.

For the SEER survival data Yu *et al* (16) describe the above model in detail. The survival times after diagnosis are grouped into intervals *I_j_* = [*t_(j-l)_t_j_*], *j*=0,1,2,..., *J* + *l*, where *t_0_*=0 and *t_J_* is the end of the follow up. In that case the hazard rate during the interval *I_j_* given that the patient is alive at the beginning of the interval is

(c) $$\lambda_{j}\left(x\right)=P\left(T<t_{j}|T\geq t_{j-1};x\right)=1-\frac{S\left(t_{j}|x\right)}{S\left(t_{j-1}|x\right)},j=1,...,J+1$$

Consider the proportional hazard model, *S*(*t_j_*)=*S_0_*(*t*)^exp{*h(x)*}^, where *S_0_*(*t_j_*) is the baseline survival function and note that

(d) $$\log\left\{-\log\left[1-\lambda_{j}\left(x\right)\right]\right\}=\log\left\{-\log\left[\frac{S_{0}\left(t_{j}\right)}{S_{0}\left(t_{j-1}\right)}\right]\right\}+h\left(x\right)$$

Denote log−logS0tj | xS0tj−1 | x+aj.

Then it is straightforward that the baseline survival function can be expressed as$S_{0}\left(t_{j}\right)=\exp\left[-\sum_{l=0}^{j}e^{a_{1}}\right]$

Under the following assumptions, and no joinpoint for the hazard function, thus *h(x)=βx* we have $\frac{\log\left[1-\lambda_{j}\left(x+1\right)\right]}{\log\left[1-\lambda_{j}\left(x\right)\right]}=\exp\left(\beta \right)$ and [exp(*β*)-1]100% can be interpreted as the annual percent change (APC) of the hazard rate, *λ_j_(x)* for the diagnosis year *x*. Accordingly, when *h(*x*)* is defined with joinpoints, the annual percent change of the hazard rate in the k-th segment will be given by [exp(*β_k_*)-1]100%.

Several authors have alternatively introduced time dependent functions (13), or discontinuous change points (7) for the baseline hazard. In this study we model the survival trend by fitting joinpoints only into the calendar year covariate following cancer diagnosis.

### Likelihood function

Survival statistics are typically expressed as the proportion of patients alive at some point after diagnosis. In order to measure the excess mortality due to the cancer of interest, we would eliminate the confounding effects of death from other causes. Relative survival is an estimate of the percentage of patients who would be expected to survive the effects of their cancer defined as the observed survival proportion divided by the expected survival rate of a comparable population who is assumed to be free of the disease of interest.

For relative survival the cause of death is not used, the adjusted number of person-years at risk *r_xj_*, by the actuarial assumption (4) is *r_xj_=n_xj_-1/2 l_xj_*, for the patient cohort diagnosed with cancer in the year *x*, where *n_xj_* is the number of people alive at the interval *I_j_*, and *l_xj_* is the number of patients lost to follow up during the beginning of interval. We will also be assuming the number of patients dying from all causes *d_xj_*, and the expected probability of surviving interval *I_j_* for the general population *E_j_(*x*)*. Usually, *d_xj_* will follow a binomial distribution

Binrxj,1−pjxEjx,pjx=1−λjx

or, in cases where the information is scarce the Poisson distribution. The likelihood function for the relative survival given in (16).

We used expected life tables calculated by the National Cancer Institute (NCI) found in (SEER Cancer Statistics Review 1975-2007), (1), the SEER*Stat analysis software was used to export survival statistics.

### Parameter estimation

Since in the present study the joinpoints are assumed to occur at observed data points, the grid search method (6) will be used to find the estimates of those points; first the log-likelihood is maximized for fixed values of *τ=(τ_1_,..., τ_*k*_)*, for a given τ the maximum value is a profile likelihood and the other associated parameters can be estimated by least squares for usual linear model. Then, all possible combinations of the joinpoints are tried by grid search and the maximum likelihood estimates of the joinpoints are the values that maximize the log-likelihood.

Asymptotic results for the joinpoint regression were proved (3). Computed confidence intervals for the location of the joinpoints using the chi-squared distribution was established. For the standard errors of the parameters as well and the resulting confidence intervals for the *β_*k*_'s*, as well as, the annual percentage changes associated (3, 13).

In practice we limit the maximum number of joinpoints to three, because we expect the gradual dissemination of the possible breakthroughs as well as few in number changes in survival. Also we restrict so that two joinpoints cannot be too close to each other and that a joinpoint cannot occur too early or too late in the study period.

### Model selection

Two basic methods for model selection are used, the Bayesian Information Criteria (BIC) and a permutation-test based approach which consists of as series of permutation tests for *H_0_:K=k_0_ vs H_1_:K=k_1_*. The first one, well established (14) and consistent when the number of true covariates does not increase with the sample size does not tend to over fit the true model like the Akaike Information Criteria (AIC). The second one, has been developed by the NCI (http://srab.cancer.gov/joinpoint) for cancer incidence and mortality rates, is carried out by permuting the residuals (5, 13, 16) and can be used to pick the joinpoint number. However, it is computationally intensive for use in survival data and that makes BIC criteria a strong competitor (13). For a K-joinpoint model *M_K_*, where *l_K_* denotes the maximum log-likelihood value for the model *M_K_*,

(e) $$\mathrm{BIC}\left(M_{K}\right)=-{2l}_{K}+p_{K}\log n$$

where n is the total number of follow-up years and *p_K_* is the number of parameters under model *M_K_*. If the possible values for the number of joinpoints is from zero to a pre-specified number, the BIC approach will select the model *M_K_* with the minimum BIC as the final model. The BIC approach has also been used to select the number of joinpoints in Bayesian models (15).

## RESULTS

### Study Cohort

We obtained survival data for individuals diagnosed with malignant brain tumor from the Public-Use Database of the SEER program, National Cancer Institute, based on the November 2009 submission (1). For patient survival analyses, only microscopically confirmed and actively followed cases were included. Patients with multiple primary tumors were excluded from these analyses.

The relative survival rate of patients with brain tumor was calculated by taking into account the expected survival of a similar cohort of the general population without the disease. The relative survival rate is the ratio of the observed survival divided by the expected survival of a cohort of the general population possessing similar characteristics with respect to age, race, sex, and era of diagnosis. The relative survival rate was calculated using SEER_Stat 6.6.2 (10), which derives expected survival rates for the general population from life tables obtained from the National Center for Health Statistics.

### Characteristics of the Participants

We identified 75,363 patients who met the study criteria. The median age at diagnosis was 72 years. The vast majority of patients in the study were white (67,501). Black patients were 4,357 and other race patients contributed 3,508 to the study cohort.

**Figure 1:**
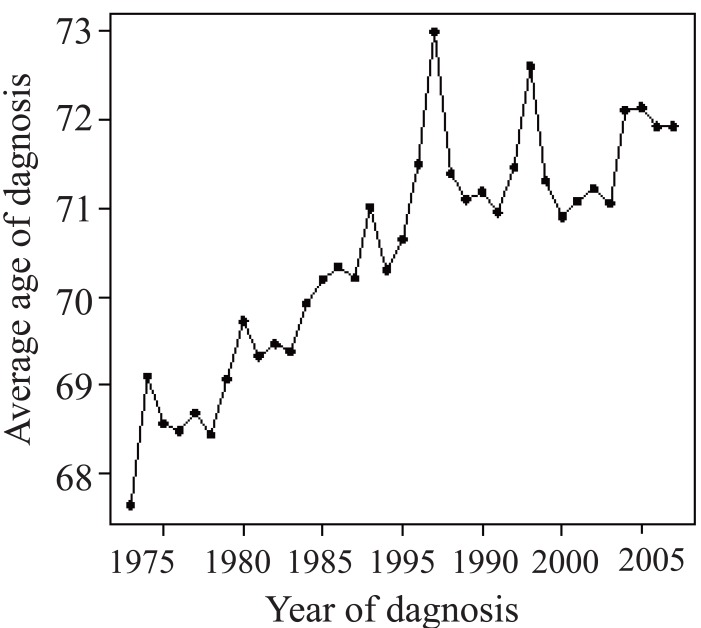
Average age of diagnosis for patients with brain cancer.

**Figure 2:**
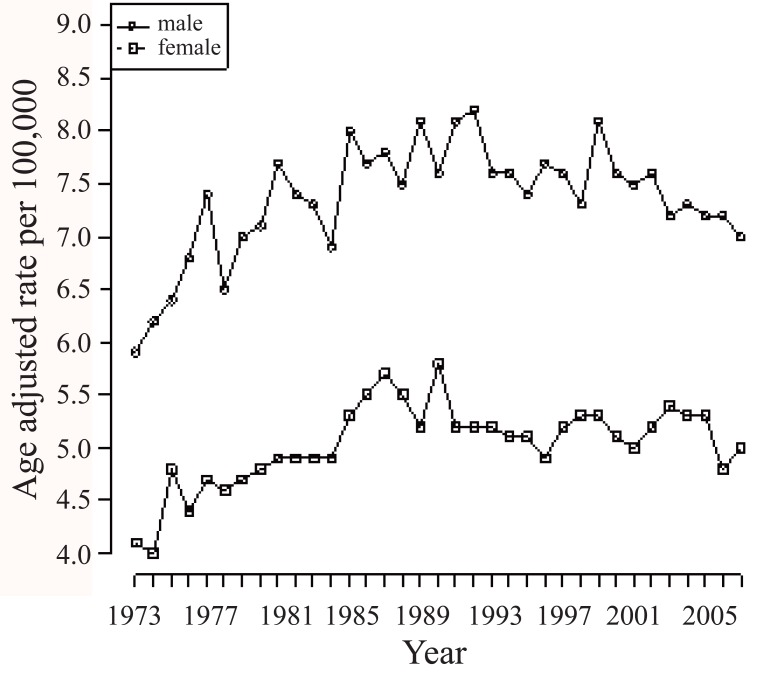
Incidence rate of males and females in brain cancer (1973, 2007).

Age may play an important role in survival trend. The average age of diagnosis of the cancer patients changes over time as shown in Figure 1. Incidence rates for brain cancer are higher in men compared with women and the gap has been unchanged in the last 30 years as shown in Figure 2.

### Statistical analysis

We compute the net (absence of other causes of death) survival rate (2) as described in Section 2, which is a key measure to assess the chance of cancer survival after diagnosis for the population. Assuming that a person may survive for many more years after being diagnosed with cancer, information on survival rates can play an important role in planning individual treatment strategies. In addition, identified differences in survival rates between subgroups of patients allow clinicians and policy makers to better target interventions.

**Figure 3:**
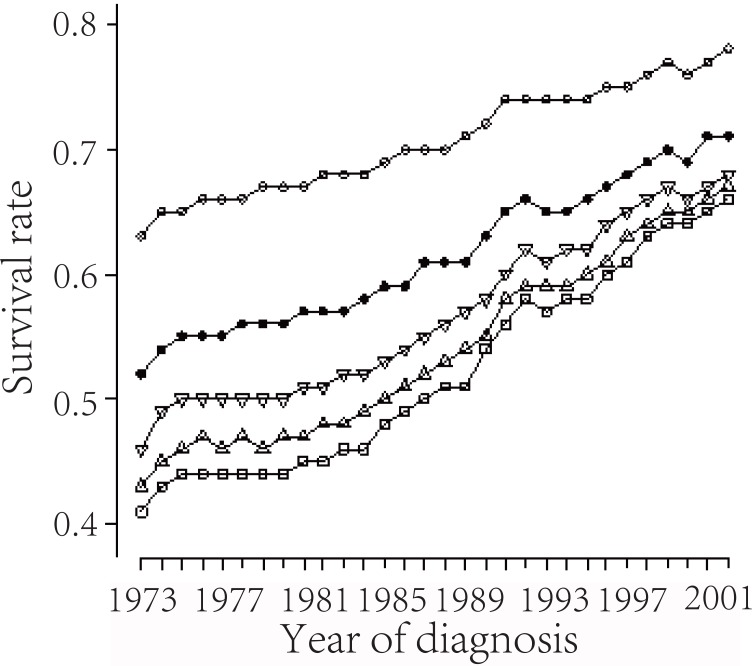
From top to bottom; 1, 2, 3, 4, 5 year relative survival probabilities plotted against the year of diagnosis.

We analyze brain cancer cases diagnosed from 1973 to 2007 with follow-up to 2007, from the SEER 17 registries, the maximum follow-up time is 35 years. The k-year actuarial survival probabilities with k=1, 2, 3, 4, 5 over the year of diagnosis, for these patients are presented in Figure 3.

We apply our models to evaluate the trend in brain cancer survival data obtained from the SEER program. To avoid the accurate specification of cause of death, in the following, we are using relative survival estimates, percentages of patients who would be expected to survive the effects of their cancer. Relative survival rates provide an estimate of patients’ survival which is corrected for competing causes of death. It is defined as the ratio of the observed survival of brain cancer patients to the expected survival of cohort matched for age, sex and geographic area.

A maximum number of joinpoints equal to 3 is employed. The primary reason for using a maximum of three joinpoints is that, cancer survival trends cannot depict too many changes in the overall trends. We use the model selection criteria already described, and compare between models, starting with no joinpoints, k=0, and moving up to k=3 joinpoints. For each value of k=0, 1, 2, 3 two different model selection methods, namely, the permutation- test-based approach, and the BIC (Bayesian Information Criteria) are employed to assess model fit, we found that the performance of the criteria are in general terms consistent in selecting the best model. We intend to show that covariates may affect the number of joinpoints.

The response variables for the analysis of relative survival are the natural logarithms of age-adjusted cancer rates. We fit the heteroscedastic/uncorrelated errors model where the variance of the errors depends on the time of the observation. The grid search uses a grid size of 1 year and the permutation tests are based on 4500 Monte Carlo replicates. We perform four permutation tests with the Bonferroni correction and an overall significance level of 0.05.

Assuming uncorrelated errors for the model to brain cancer relative survival data we obtained *p*-values of 0.012 testing the null hypothesis of 0 joinpoints against the alternative of 4 joinpoints, 0.016 testing 1 joinpoint against 2 joinpoints and 0.016 testing 1 joinpoint against 3 joinpoints. Comparing these to the critical value of 0.05/2, we reject all three null hypotheses and therefore select the one-joinpoint model as our final model. Fitting the same model using BIC as our selection criteria for the best fit model we concluded that it would be one with two joinpoints (one on 1989 and one on 1998). The resulting estimates are shown in Table 1, reflecting on the conclusive evidence against the statistical significance of one of the parameters we will reject this model as our final candidate.

**Table 1:** Possible joinpoint model estimates for the SEER brain cancer data

Parameter	Parameter Estimate	Standard Error	Prob > |t|

Intercept 1	3.711836	0.023503	0.000000
Intercept 2	3.958221	0.092750	0.000000
Intercept 3	3.659534	0.073028	0.000000
Slope 1	0.014971	0.002339	0.000001
Slope 2	-0.000428	0.004437	0.923931
Slope 3	0.011520	0.002371	0.000045

**Figure 4:**
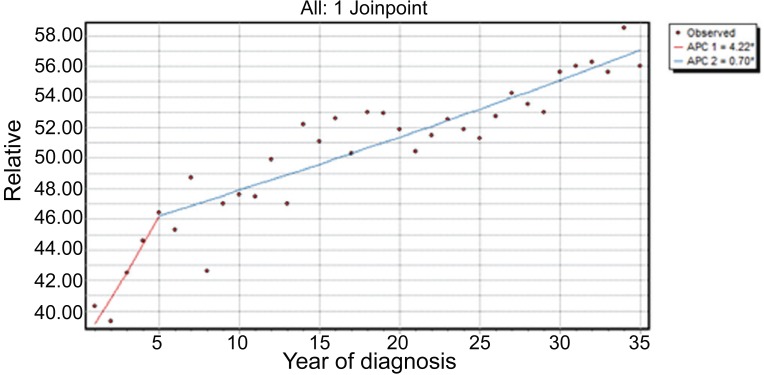
The proposed joinpoint line fit for the relative 12 month survival rate (in %) of brain cancer patients over the years 1973 to 2007 along with the observed values. On the top right, the annual percentage changes experienced in survival under the model during the two periods involved.

A Poisson error model which departs from the assumption of normality for the errors also suggested the one joinpoint model regardless of the best fit model criteria; both suggested the relative model as the best choice. Figure 4 shows the observed data and the final one-joinpoint at 1978 model under the uncorrelated model and in Table 2 we present the estimates of the model coefficients along with their standard errors.

**Table 2:** Final joinpoint model estimates for the SEER brain cancer data

Parameter	Parameter Estimate	Standard Error	Prob > |t|

Intercept 1	3.627167	0.044683	0.000000
Intercept 2	3.799012	0.015608	0.000000
Slope 1	0.041369	0.016040	0.015053
Slope 2	0.007000	0.000687	0.000000

**Figure 5:**
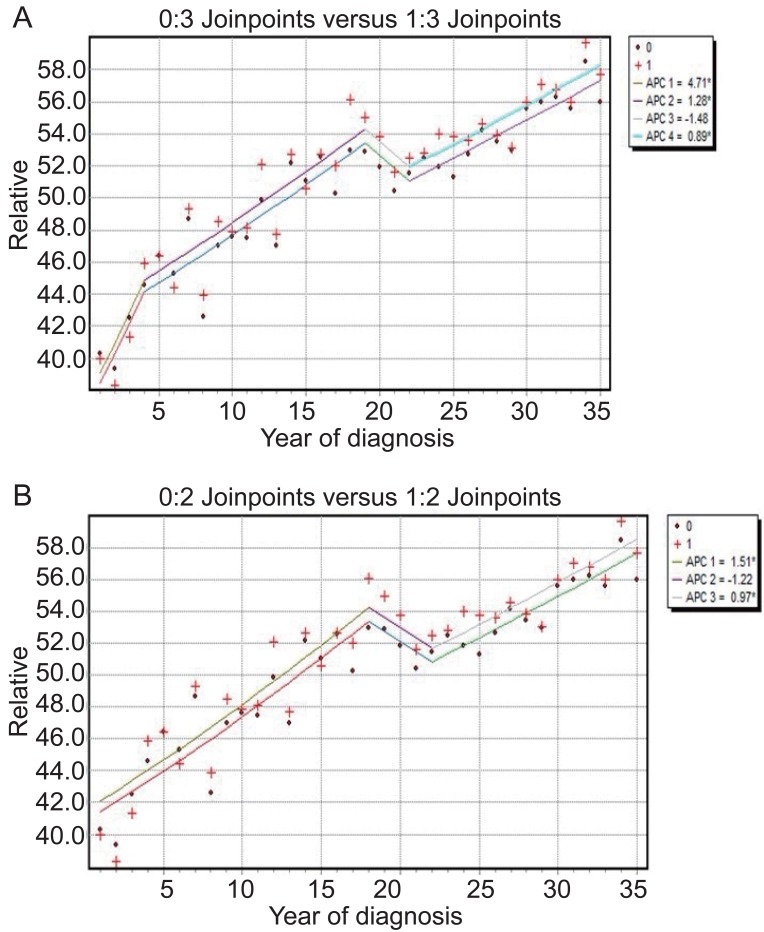
Brain cancer relative survival rates: (A) Poissson uncorrelated errors model (3 joinpoints) with APC printed for each line segment; (B) Normal errors model (2 joinpoints) with APC. Coded with zero is the general population and coded with 1 is the male population.

In an effort to test weather the temporal survival improvements were not observed uniformly across the two sexes we fitted individual by-sex models and compared them with the general model. The results were quite encouraging about the homogeneity of the experienced survival improvement across different sexes, as shown in Figure 5, were the overall model is plotted along the male sub model. The same results were found by the comparison of the other resulting pair (female- mixed). Our conclusion is firm that the resulting lines are parallel and this was confirmed by formal statistical tests which we do not report here.

Figure 5(*a*) shows the observed data and the final three-joinpoint model under the Poisson model with joinpoints at 1977, 1992 and 1995. Figure 5(*b*) shows the final two-joinpoint model under the normal errors model with joinpoints at 1991 and 1995. Comparing the two models we see that they are quite similar, except that the two-joinpoint model ignores a slight change in trend in the 1973-1977 interval.

## DISCUSSION

We followed a well-documented procedure to identify changes in trend data for brain cancer patients in the USA. In the process of comparing cancer survival with special interest in recent years we would like to stress the following: the joinpoints once identified should not become fixed as we search for additional ones in future circumstances. The procedure employed here identified the best fitting set of points over the entire data in hand. There have been instances in the literature (17) where sequential tests were used. The joinpoints once identified, become fixed as we search for additional ones. Since the 27 years of data have already been observed, it seems better to analyze the overall best fit. In future years, however, we should be cautious in maintaining a valid model, in the presence of repeated analysis, adding on additional point at a time.

Bayesian methods as mentioned before (13) have also been applied to joinpoint regression for cancer rates. Those approaches will incorporate a prior distribution on the number of joinpoints, the other regression parameters and the error variances. The estimation of the marginal likelihoods is based on the Markov chain Monte Carlo (MCMC) method, described in (5).

The model selection criteria employed, especially BIC, tend to detect a lower number of joinpoints compared with AIC or the permutation tests approach. To detect the accuracy of those models it would be useful to compare their estimates with those resulting from the standard survival models.
